# Effects of Resistance Training Prior to Total Hip or Knee Replacement on Post-operative Recovery in Functional Performance: A Systematic Review and Meta-Analysis

**DOI:** 10.3389/fspor.2022.924307

**Published:** 2022-07-14

**Authors:** Stian Langgård Jørgensen, Signe Kierkegaard, Marie Bagger Bohn, Per Aagaard, Inger Mechlenburg

**Affiliations:** ^1^Department of Occupational and Physical Therapy, Horsens Regional Hospital, Horsens, Denmark; ^2^Horsens Research Centre - Hip Training & Preservation Surgery (H-HIP), Department of Occupational and Physical Therapy, Horsens Regional Hospital, Horsens, Denmark; ^3^Department of Orthopedic Surgery, Horsens Regional Hospital, Horsens, Denmark; ^4^Department of Clinical Medicine, Aarhus University, Aarhus, Denmark; ^5^Department of Sports Science and Clinical Biomechanics, University of Southern Denmark, Odense, Denmark; ^6^Department of Orthopedic Surgery, Aarhus University Hospital, Aarhus, Denmark

**Keywords:** prehabilitation, functional performance, muscle strength, orthopedics, patient reported outcomes

## Abstract

**Objective:**

To evaluate the effectiveness of pre-operative resistance training in patients allocated to TJR surgery on selected post-operative outcomes, *via* a meta-analysis of studies using exercise modalities and loading intensities objectively known to promote gains in muscle size and strength in adults of young-to-old age.

**Design:**

A systematic review and meta-analysis.

**Literature Search:**

Cochrane Central, MEDLINE, EMBASE, and PEDro were searched on August 4th 2021.

**Study Selection:**

Randomized Controlled Trials (RCTs) were included if (i) they compared pre-operative lower-limb-exercises before elective TJR with standard care, (ii) explicitly reported the exercise intensity, and (iii) reported data on functional performance.

**Data Synthesis:**

This systematic review and meta-analysis is reported in accordance with the PRISMA reporting guidelines. A random effects model with an adjustment to the confidence interval was performed for pooling the data.

**Results:**

One thousand studies were identified. After applying exclusion criteria, five RCTs were located including 256 participants (mean age ranged from 61 to 72 years, 54% women). Moderate-to-large improvements in functional performance and maximal knee extensor strength were observed at 3 months after surgery along with small-to-moderate effects 12 months post-operatively. For patient-reported outcomes, small-to-moderate improvements were observed at 3 months post-operatively with no-to-small improvements at 12 months.

**Conclusion:**

Prehabilitation efforts involving progressive resistance training provides an effective means to improve post-operative outcomes related to functional performance, knee extensor strength and patient-reported outcome in patients undergoing TJR. Due to large methodological diversity between studies, an optimal loading intensity remains unknown.

**Systematic Review Registration:**

Prospero ID: CRD42021264796.

## Introduction

Total knee or hip replacement surgery (TJR) is typically offered to patients to reduce joint pain and increase quality of life (Skoffer et al., 2015; Moyer et al., 2017). However, up to 20% of the patients report a non-optimal outcome after surgery due to insufficient pain relief and/or persistent deficits in functional capacity (Moyer et al., 2017; Franz et al., 2018). In fact, functional performance and muscle strength have been observed to remain below levels of healthy age-matched adults even years after surgery (Mizner et al., 2005; Bade et al., 2010).

The prevalence of TJR procedures is increasing internationally (Moyer et al., 2017; Odgaard et al., 2020a,b) with an projected growth of 174% THR- and 673% TKR-procedures from 2005 to 2030 in the United States alone (Kurtz et al., 2007). Thus, efficient and safe treatment paradigms seem highly warranted.

Preoperative exercise-based training (prehabilitation) has been suggested as an essential component, attributing to a fast recovery after TJR (Franz et al., 2018; Ghosh and Chatterji, 2018; Lim and Thahir, 2021). However, often with limited pre-operative time from accepting surgery to the day-of-surgery (i.e., 4–12 weeks), it is reasonable to address impairments expected to have significant impact on the post-operative outcome, such as lower limb muscle mass and strength. In support of this notion, both pre-operative functional performance and lower limb muscle strength have each been positively associated with post-operative functional performance up to 2 years post-operatively in patients receiving TJR (Fortin et al., 1999; Bade et al., 2010; Zeni and Snyder-Mackler, 2010; Holstege et al., 2011; Nankaku et al., 2013; Skoffer et al., 2015). Thus, despite involving different surgical procedures and affecting different muscle groups, both patient populations (TKR, THR) are likely to benefit from exercise interventions that aim at increasing lower limb muscle strength and improving functional performance.

Progressive resistance training (PRT) is commonly referred to as the Gold Standard intervention modality for promoting consistent gains in mechanical muscle function in healthy individuals (Garber et al., 2011). Also, PRT is often applied in older adults and selected orthopedic populations using loading intensities ranging from ~60–85% 1 repetition maximum (1 RM), typically leading to substantial improvements in functional performance, muscle strength and muscle hypertrophy (Suetta, 2004; Aagaard et al., 2010; Steib et al., 2010; Borde et al., 2015; Csapo and Alegre, 2016; Skoffer et al., 2016; Ferraz et al., 2018; Hughes et al., 2019). Notably, novel training approaches using lower exercise loads and concurrent restriction of blood flow to the exercising limb such as low-load blood flow restricted exercise appear effective also regarding increasing skeletal muscle strength and improving functional performance in patients suffering from knee OA (Segal N. A. et al., 2015; Segal N. et al., 2015; Bryk et al., 2016; Ferraz et al., 2018).

Previous systematic reviews and meta-analyses have generally observed no-to-little evidence in favor of prehabilitation after TJR surgery (Kwok et al., 2015; Wang et al., 2016; Chesham and Shanmugam, 2017; Husted et al., 2020) with the overall evidence of moderate-to-low methodological quality (Kwok et al., 2015; Skoffer et al., 2015; Chesham and Shanmugam, 2017; Moyer et al., 2017). However, no restrictions on the specific loading/exercise intensity (%1 RM) employed in these reviews (Kwok et al., 2015; Skoffer et al., 2015; Wang et al., 2016; Chesham and Shanmugam, 2017; Moyer et al., 2017; Husted et al., 2020). To remove the noise from exercise interventions of insufficient (i.e., too low) exercise intensities, the present systematic review and meta-analysis aimed to evaluate the effectiveness of pre-operative training in patients scheduled for elective TJR using exercise modalities and loading intensities objectively known to promote gains in muscle size and strength in the spectrum of healthy populations of young-to-old age.

## Methods

The review was conducted in accordance with PRISMA statement guidelines (Moher et al., 2015) and was registered at the International Prospective Register of Systematic Reviews (PROSPERO): CRD42021264796.

### Search Strategy

A literature search was conducted at the Cochrane Central Register of Controlled Trials, MEDLINE, EMBASE, and the Physiotherapy Evidence Database on August 4th 2021. As our institutions did not hold the rights to complete searches in The Allied and Complementary Database, it was not possible to accommodate this element of the PROSPERO protocol.

Search terms are presented in Supplementary File 1.

Two authors (SLJ, SK) independently screened titles and abstracts to identify potentially eligible trials based on predetermined criteria. The full text of potentially eligible papers was retrieved and independently assessed by the same two authors to determine eligibility. Any disagreements were resolved *via* consensus or by consulting a third author (IM) when necessary.

### Eligibility Criteria

Studies were eligible for inclusion in the present meta-analysis if fulfilling the following criteria: (i) involving a randomized controlled trial (RCT) design, (ii) written in English, (iii) comparing the post-operative effect of pre-operative lower-limb PRT exercise performed prior to TJR to usual care or control interventions, (iv) containing specific information about the exercise intensity, and (v) including data on functional performance. Trials were excluded if: not designed as a RCT, including participants scheduled for TJR for other reasons than OA (i.e., rheumatoid arthritis or trauma), or if not reporting exercise intensity for the intervention group(s).

### Inclusion Exclusion Criteria

Inclusion criteria were trials that used exercise paradigms designed and implemented to increase lower limb muscle strength and promote skeletal muscle hypertrophy (Steib et al., 2010; Borde et al., 2015; Patterson et al., 2019). Before initiating the literature search, we specified the original criteria outlined in our PROSPERO registration protocol to comprise studies utilizing (i) resistance exercises with loading intensities ≥60% 1 RM, (ii) resistance training employing moderate-to-low load intensities (<60% 1 RM) performed to concentric contraction failure in at least the final exercise set in each exercise, or (iii) exercising with low loads and concurrent blood flow restriction for the exercising limb (Kim et al., 2017).

Co-interventions, including patient education, mobilization, manipulation, massage therapies, glucocorticoid injection, analgesia, balance training, knee and hip joint mobility exercises were allowed, except if dose/exposure was distributed unequally between the intervention and control groups, in which case studies were excluded from the analysis.

### Comparator Groups

Included studies were allowed to use control groups allocated to usual care or control interventions.

### Outcome Measures

To assess the effectiveness of the pre-operative intervention procedures, the present meta-analysis included the following functional performance tests: (i) Sit-to-stand tests, (ii) Ambulatory function assessed by the Timed Up & Go test (Alghadir et al., 2015), (iii) Stair climbing test, (iv) habitual horizontal walking speed, and (v) maximal isometric voluntary knee extensor muscle strength assessed either using isokinetic dynamometry or hand held dynamometry (Aagaard et al., 2002; Koblbauer et al., 2011).

Duration of time to follow-up was characterized as medium-term (2–4 months) or long-term (10–12 months or longer). If a study reported both medium-term and long-term outcome data, data from both time points were extracted. If the same RCT divided the reporting of medium-term and long-term follow-up data into separate publications, the results were used separately in the relevant meta-analysis. Further, functional tests measuring time required to perform a pre-set number of repetitions (i.e., 5 times sit-to-stand test) were converted into repetitions per second to allow a standardized analysis, with increasing values representing enhanced test performance in all cases. The specific conversion was performed using the following equation on the raw dataset [online Supplementary Material (Holsgaard-Larsen et al., 2020)]: $\frac{Repetitions\;\left(reps\right)}{Time\;\left(t\right)}$.

### Quality Assessment

**Figure 1:**
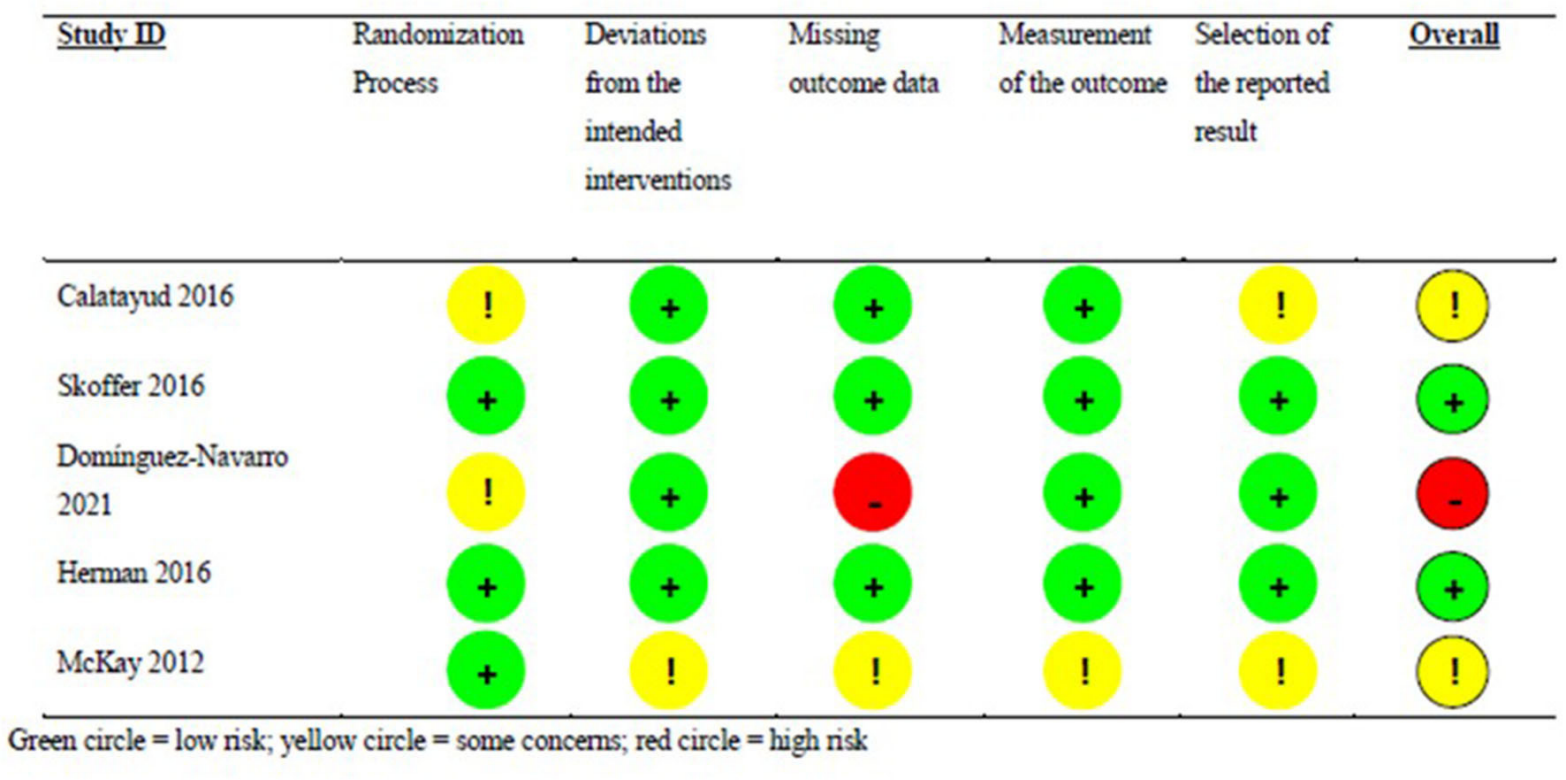
Risk of Bias Assessment for each individual study. Green circle, low risk; yellow circle, some concerns; red circle, high risk.

Risk of Bias (RoB) assessments (Figure 1) were performed using the Cochrane Collaboration's tool for assessing RoB, as described in detail previously (Higgins et al., 2019). The RoB assessment scores on the reporting of judgement items were: (i) Adequate (*Bias, if present, is unlikely to alter the results seriously)*, (ii) Unclear *(A risk of bias that raises some doubt about the results)*, and (iii) Inadequate *(Bias may alter the results seriously)*, corresponding with (i) low, (ii), unclear, and (iii) high risk of bias, respectively (Grønfeldt et al., 2020).

The RoB analysis included five distinct aspects of reporting: the randomization process, deviations from the intended intervention, missing outcome data, measurement of the outcome variables, and selected reporting of the obtained results.

RoB was performed independently by two reviewers (SJ, IM) and discrepancies were resolved through discussion until reaching consensus. As IM coauthored Skoffer et al. (2016, 2020), the RoB assessment was performed by SJ and PA.

The Grades of Recommendation, Assessment, Development and Evaluation (GRADE) scheme (Atkins et al., 2004; Guyatt et al., 2008) was used to assess the quality of evidence in the performed meta-analyses. The overall GRADE certainty ratings include “very low,” “low,” “moderate,” and “high” (Brignardello-Petersen et al., 2018).

## Data Extraction, Synthesis, and Analysis

Two authors (SJ, SK) both extracted data from each study by following a predefined scheme. Data were cross-checked for differences in data-extraction and discrepancies were resolved through discussion until agreement was reached. Otherwise, a third author was consulted until consensus was reached (MB).

The following data were extracted from each study:

1. Trial characteristics (sample size, first author name, year of publication, type of trial, country, source of funding, trial registration status, reported sources of bias/conflicts of interest).
2. Participant characteristics (inclusion and exclusion criteria, age, sex, body mass).
3. Intervention procedures, including exercise.
4. Comparator/control group intervention, exercise characteristics if applicable.
5. Co-interventions, if any, reported for each group.
6. Outcomes variables reported, including time of assessment.

Due to the small number of included trials, the meta-analyses were performed using a random effects model with an adjustment to the confidence interval proposed by IntHout et al. (2014) computing the effect size (Hedges' g) of the included prehabilitation intervention protocols compared with their respective control group (Higgins et al., 2019). Results were extracted in form of post-intervention group mean data, standard deviation (SD) and sample size as inputs for the meta-analyses. In case of incomplete data, means and SDs were extrapolated from article graphs (WebPlotDigitizer 4.5).

As we assume outcome variables to be in collected in different units across studies, data are presented as standardized mean difference (SMD) along with their respective 95% confidence intervals (CI). For interpretation of the SMD, the following definitions were adopted: >0.2 small effect, >0.5 moderate effect, >0.8 large effect (Cohen, 2013).

Heterogeneity between the included studies was assessed using the *I*^2^ statistics and interpreted as low (*I*^2^ = 0–30%), moderate (*I*^2^ = 30–60%) and high (*I*^2^ ≥ 60%) heterogeneity (Higgins et al., 2003, 2019). All statistical analyses were conducted in Stata 17.0 (StataCorp, TX, USA).

## Results

### Summary of Findings

**Figure 2:**
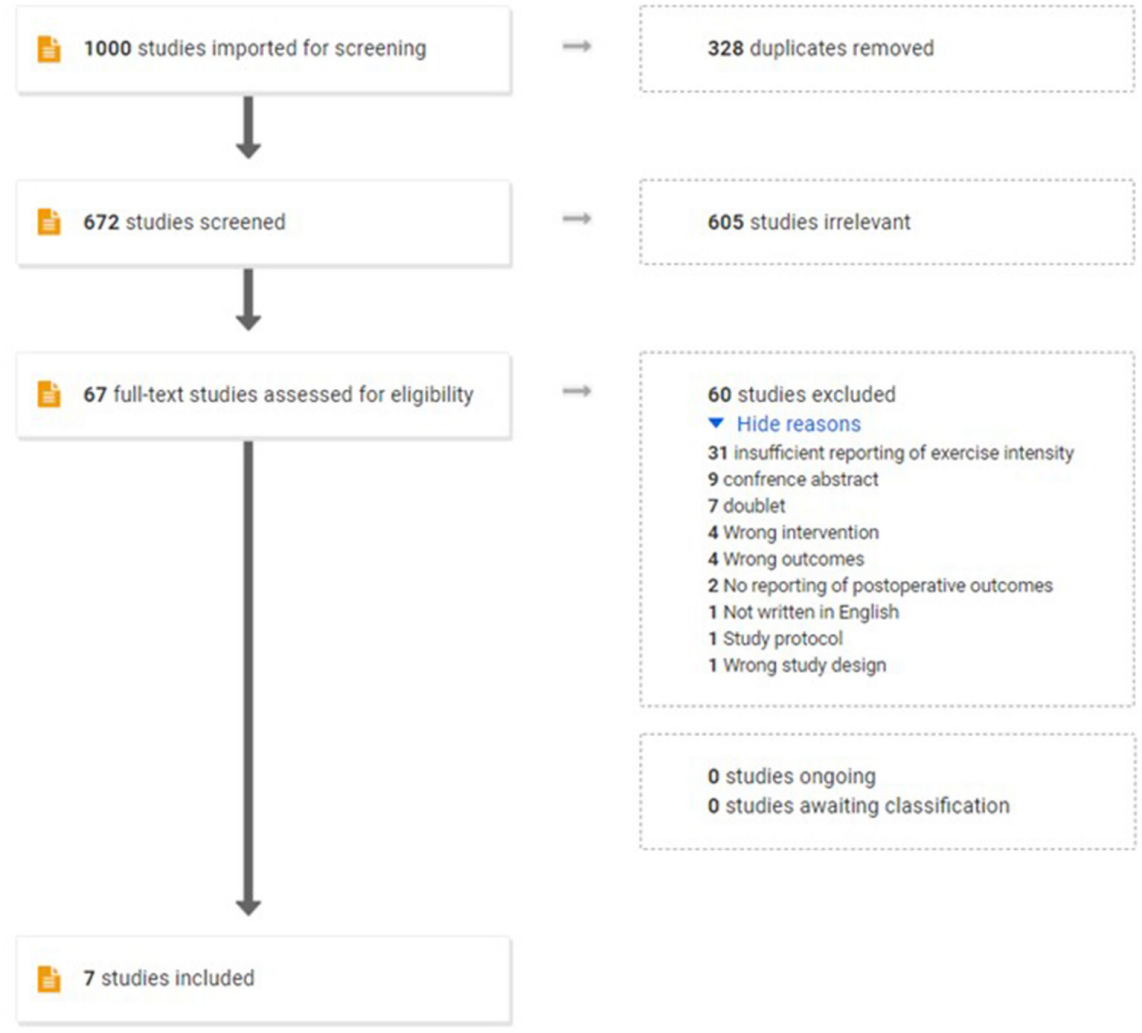
Flow chart of the study selection process.

We identified 1,000 hits from the literature search performed on August 4th 2021. After removing duplicates, 672 potentially eligible trials were identified (Figure 2). Following title and abstract screening, 605 records were excluded while 66 records remained for full-text reading. A total of 59 records were excluded for not meeting the inclusion criteria, leaving a total of seven studies to be included in the present analysis. Four trials reported baseline and short-term follow-up data on patients scheduled for TKR: McKay et al. (2012), Calatayud et al. (2016), Skoffer et al. (2016), and Domínguez-Navarro et al. (2021) and a single study reported baseline and short-term follow-up data on patients scheduled for THR: Hermann et al. (2015). In addition, two articles reported long-term follow-up data based on the above studies, namely: Skoffer et al. (2020) [follow-up data based on Skoffer et al. (2016)] and Holsgaard-Larsen et al. (2020) [follow-up data based on Hermann et al. (2015)]. Ultimately, seven papers were deemed eligible in the present meta-analyses. However, since each study trial only could be counted once in each seperate analysis, a maximum of five trials per analysis was possible. Data from Hermann et al. (2015) and Holsgaard-Larsen et al. (2020) were extracted from available Supplementary Spread Sheet Files (Holsgaard-Larsen et al., 2020).

According to our preregistered Prospero protocol (https://www.crd.york.ac.uk/prospero/display_record.php?RecordID=264796), it was the explicit study aim to investigate the effect of pre-operative resistance training (PRT) on the post-operative recovery following total knee replacement (TKR) as well as hip replacement (THR). To adhere to the Prospero protocol, we have retained the single trial on THR in our analysis.

### Trial, Participants, and Intervention Characteristics

Individual study characteristics are summarized in Table 1. A total of 256 patients scheduled for TJR were included in the meta-analysis (176 TKR/80 THR). Mean age was 61–72 years and 54% of the participants were women. Four trials provided usual care (Hermann et al., 2015; Calatayud et al., 2016; Skoffer et al., 2016; Domínguez-Navarro et al., 2021) while one trial involved control intervention (McKay et al., 2012) in the control group. Two trials utilized a percentage of 1 RM to quantify training load intensity (McKay et al., 2012; Domínguez-Navarro et al., 2021), while three trials controlled loading intensity by adjusting exercise loads to target a certain number of repetitions when performed to contraction failure (Hermann et al., 2015; Calatayud et al., 2016; Skoffer et al., 2016). Training periods ranged from 4 to 10 weeks 2–3 times per week. All exercise sessions were supervised in all trials. Baseline, 3- and 12-month follow-up assessments for all included studies are reported in Table 2. As all medium-term data were collected at 3 months post-operatively and all long-term data were collected at 12, 3, and 12 months were used in the following sections as temporal terms to denote “medium-term” and “long-term” effects, respectively. Only Holsgaard-Larsen et al. (2020) reported findings 5–7 months post-operatively. Therefore, we decided to exclude this intermediate time point from the present analysis. Also, stair climbing performance, knee flexor strength, and hip extensor and flexor strength were reported by a single study only at 12 months post-operatively. Therefore, these time points for these particular outcome variables were excluded from the present long-term (12 month) analysis. None of the studies assessed markers related to skeletal muscle mass.

**Table 1:** Trial characteristics of the five included randomized controlled trials.

**References**	**Sample size Design Country Trial registrations**	**Inclusion criteria Exclusion criteria**	**INT *n*, (female) CON *n*, (female %)**	**INT** **Mean age (SD) Mean body mass (SD) Mean height (SD) Mean BMI (SD)** **CON** **Mean age (SD) Mean body mass (SD) Mean height (SD) Mean BMI (SD)**	**Exercise intervention Duration Frequency/week** **INT** **Exercises Duration Frequency/week**	**INT** **Contraction mode Exercise intensity** **Set** **Repetitions Rest between sets Training progression Supervision** **CON**	**Post-operative rehabilitation**	**Outcomes variables**
Skoffer et al. (2016)	59 Assessor-blinded, clinical randomized controlled trial Denmark NCT01647243	Patients scheduled for TKA, were radiographically and clinically diagnosed with OA, were residents in the Aarhus municipality, and were able to transport themselves to training Patients below 18 years of age, had heart disease or uncontrolled hypertension, had neuromuscular or neurodegenerative conditions, or were unable to comprehend the protocol instructions	30 (11) 29 (17)	**INT** 70.7 yrs (7.3) 83.6 kg (median) (range: 56.8–117.2) 167 (median) (range: 145–184) 30.0 (kg/m^2^) (median) (range: 22.6–42.5) **CON** 70.1 yrs (6.4) 91.9 kg (median) (range: 66.2–137.4) 170 (median) (range: 146–197) 31.8 (kg/m^2^) (median) (range: 24.3–42.2)	INT Leg press, knee extension, knee flexion, hip extension, hip abduction, hip adduction 4 weeks 3/week **CON** Post-operative PRT identical to the PRT applied to the intervention group pre/post surgery	Concentric + eccentric contractions 12 to 8 RM 3 sets 8–12 reps 120 s Progressed from 12 RM toward 8RM, with no further information on the progression All PRT training session took place at Aarhus University Hospital and were supervised by 3 physiotherapists specially trained in the applied training concept. **CON** No information	Both INT and CON performed 4 weeks of PRT identical to the pre-operative PRT protocol	30 s chair stand test (repetitions) Timed Up & Go (seconds) 10-m walk test (seconds) Isometric knee extension (Nm/Kg) KOOS Pain KOOS Symptoms KOOS ADL KOOS Sport & Recreation KOOS QOL
Hermann et al. (2015)	80 Prospective, randomized controlled trial Denmark NCT01164111	Diagnosed with primary hip OA aged 50 years or older, scheduled for THA at the Department of Orthopedic Surgery, Herlev University Hospital, Copenhagen, Denmark Rheumatoid arthritis and other types of arthritic not diagnosed as OA, uraemia, cancer, treatment with systemic glucocorticoids >3 months the last 5 years with a dose ≥ 5 mg, present or previous hip fracture (either side), other lower extremity fracture wi	**INT** 40 (27) **CON** 40 (25)	**INT** 70.0 yrs (7.7) 78.3 kg (16.4) 167 cm (9) 28.2 (kg/m^2^) (5.3) **CON** 70.8 (7.5) 76.5 kg (7.5) 167 cm (10) 27.4 (kg/m^2^) (3.8)	**INT**' Hip extension performed in forward standing position, knee extension, knee flexion, leg press (Performed in a random circle, unilaterally) 10 weeks 2/week **CON** Usual care	The participants performed the concentric phase of the movement “as fast as possible,” and eccentric phase in ~2–3 s 8–12RM 3 set 8–12 reps No information on rest period The participants were encouraged to perform the maximum number of repetitions possible within each series. If the umber was below 8 or exceeded 12, the loading was adjusted by experienced physiotherapists. Training was performed in groups of up to 8 participants supervised by 2 physiotherapists	There were no restrictions in engaging exercise programs outside the study for any of the groups and usual care was provided during rehabilitation. In short patients were mobilized immediately after surgery with full weight-bearing and no movement restrictions and were offered supervised low-intensity rehabilitation twice a week for 4–6 weeks	HOOS ADL HOOS Pain HOOS Symptoms HOOS Sport & Recreation HOOS QOL Star ascend (seconds) Chair rise (seconds) Gait 25 meter. max speed (seconds) Isometric knee extension (Nm)
Calatayud et al. (2016)	44 RCT Spain	If pain was present in the contralateral limb (minimum pain ≥4 of 10 during daily activities), if they had undergone another hip or knee replacement in the previous year, if they had any medical conditions in which exercise was contraindicated, or if they	**INT** 25 (21) **CON** 25 (22)	**INT** 66.8 yrs (4.8) 82.1 kg (11.8) 160 cm (10) 32 (kg/m^2^) (4.2) **CON** 66.7 yrs (3.1) 80.9 kg (9.9) 160 cm (10) 31 (kg/m^2^) (3.8)	**INT** Seated leg press, knee extension, leg curl, hip abduction 8 weeks 3/week **CON** Usual care	Concentric + Eccentric 10 RM 5 sets 10 reps 60 s No information on progression Each training took place under supervision of an experienced physical therapist	After TKA surgery, all subjects received the same post-operative rehabilitation protocol at the hospital as a part of the usual care treatment. This programme was focused in restoring knee ROM, strength and normal gait. The strength exercises were specially focused on knee extensor strength, starting without external load and progressing by adding a maximum of 2 or 3 kg. Manual therapy, proprioceptive training and ice were also applied after the strength training. This rehabilitation programme was daily performed (from Monday to Friday) during 1 month, and each session lasted 1 h. The physiotherapist conducting this rehabilitation protocol was not involved in any assessment performed during the present study	Knee range of motion, flexion (degree) Knee range of motion, extension (degree) Timed Up & Go (seconds) Stair test (seconds) Isometric knee extension (kg) Womac, Pain Womac, function
Domínguez-Navarro et al. (2021)	82 Prospective randomized controlled trial with three arms Spain NCT02995668	On the waiting list for primary TKR, referred by the surgeon, were aged between 60 and 80 years, presented with advanced idiopathic knee osteoarthritis with a score of >3 in the Kellgren-Lawrence scale, and were scheduled with sufficient time until surgery Cognitive or physical baseline status that prevented patients from safely participating in the assessments and/or interventions, which corresponded to scores (1) lower than 20 in the Spanish version of the Mini-Mental State Examination and (2) lower than	**INT** 24 (10) **CON** 21 (7)	**INT** 76.9 kg (7.3) 162.2 cm (4.7) **CON** 77.4 kg (8.3) 161.3 cm (6.5)	**INT** Active leg raise^*^, Seated Knee Extension, Seated Knee flexion, Lateral Abduction^**^, Adduction^**^ 4 weeks 3/week **CON** Usual care	**INT** Concentric + Eccentric Set 1: 50%10 RM Set 2: 75%10 RM Set 3: 100%10 RM (if possible) ^*^50%10 RM. No progression ^**^No progression 3 sets ^*^5 min ^**^ 5 min 10 reps 60 s Progression from 50% 10RM to 75% 10 RM and 10 RM was performed if possible. Otherwise, the load applied was the maximum the participants could stand Supervised **CON** No information on usual care	After discharge, the participants were scheduled to perform 12 sessions of standard outpatient rehabilitation, which started 10–12 days after surgery. The protocol was supervised by a physiotherapist blinded to the group allocation results and not involved in the outcome assessment	Isometric knee extension (N) Timed Up & Go (seconds) KOOS ADL KOOS Symptoms KOOS Pain KOOS QOL
McKay et al. (2012)	22 2-arm, parallel, randomized controlled pilot trial United States of America	Had a primary diagnosis of knee OA, were ambulatory with or without a walking aide, and exhibited unilateral or bilateral OA symptoms Had scheduled additional, unrelated surgery within 3 months of their TKA, had undergone surgery in the 3 months before recruitment, had contraindications for exercise, or were undergoing a revision surgery	**INT** 10 (5) **CON** 12 (8)	**INT** 63.58 yrs (4.93) 33.78 (kg/m^2^) (7.05) **CON** 60.58 yrs (8.05) 33.05 (kg/m^2^) (6.13)	**INT** Calf raise^*^ Leg press Knee extension Leg curl 6 weeks 3/week **CON** Lat(issimus dorsi) pull Chest press Elbow flexion Elbox extension 6 weeks 3/week	**INT** Concentric + Eccentric 60%1 RM bodyweight 2 sets 8 reps No information on rest between sets 1:1 supervision by a trained kinesiologist during each of their sessions Increasing gradually with 1–2 kg. per week as tolerated, over the course of the 6-weeks The same exercise variable was applied for the CON group	All of the participants received standard post-operative care from a single physiotherapist through the hospital-based program	Isometric knee extension (Nm/kg) 50-foot walking test (seconds) Stair test (seconds) Womac Pain Womac Function

* *Dose: 3 sets at 50%10RM. No progression*;

** *Dose: 5 minutes. No progression*.

**Table 2:** Outcome variables from each individual trial.

**Domain**	**Study**	**Assessment method**	**Outcome variable**		**Intervention group: Pre-operative 3 months post-operative 12 months post-operative (Mean ±SD)**	**Control group: Pre-operative 3 months post-operative 12 months post-operative (Mean ±SD)**
Sit-to-stand	Skoffer et al., 2016, 2020	30-s sit-to-stand	Repetitions	Baseline	10.8 ± 5.1	10.4 ± 3.3
				3 m post	14.7 ± 4.7	11.0 ± 4.4
				12 m post	14.7 ± 3.8	13.1 ± 3.1
Sit-to-stand	Hermann et al., 2015; Holsgaard-Larsen et al., 2020	5 times sit-to-stand	Seconds	Baseline	14.5 ± 5.4	15.1 ± 6.9
				3 m post	9.4 ± 1.8	13.5 ± 7.6
				12 m post	9.6 ± 3.5	12.0 ± 5.5
Ambulatory function	Skoffer et al., 2016, 2020	Timed Up & Go	Seconds	Baseline	9.1 ± 2.6	9.3 ± 3.0
				3 m post	7.9 ± 2.3	8.9 ± 2.1
				12 m post	7.5 ± 2.2	7.7 ± 1.6
Ambulatory function	Calatayud et al., 2016	Timed Up & Go	Seconds	Baseline	8.6 ± 0.8	8.5 ± 0.8
				3 m post	7.0 ± 0.7	8.7 ± 1.0
				12 m post	N/A	N/A
Ambulatory function	Domínguez-Navarro et al., 2021	Timed Up & Go	Seconds	Baseline	16.1 ± 10.2	15.6 ± 5.8
				3 m post	N/A	N/A
				12 m post	11.1 ± 3.1	12.1 ± 2.9
Walking speed	Skoffer et al., 2016, 2020	10 m walking test	Seconds	Baseline	7.7 ± 1.8	7.9 ± 1.5
				3 m post	7.1 ± 1.5	7.7 ± 1.2
				12 m post	6.7 ± 1.3	7.0 ± 1.1
Walking speed	Hermann et al., 2015; Holsgaard-Larsen et al., 2020	25 m maximal speed	Seconds	Baseline	13.8 ± 3.9 ± 3.9	14.7 ± 4.5
				3 m post	11.2 ± 1.7 ± 1.7	13.6 ± 3.7
				12 m post	11.6 ± 2.6 ± 2.6	13.4 ± 4.9
Walking speed	McKay et al., 2012	50 feet walk test	Seconds	Baseline	16.88 ± 16.14	14.21 ± 5.36
				3 m post	11.80 ± 5.66	11.82 ± 2.97
				12 m post	N/A	N/A
Stair test	Holsgaard-Larsen et al., 2020	Stair ascent	Seconds	Baseline	7.2 ± 3.7	7.1 ± 3.7
				3 m post	4.8 ± 1.6	6.5 ± 4.1
				12 m post	4.6 ± 1.5	6.5 ± 4.1
Stair test	Calatayud et al., 2016	Stair ascent/descent	Seconds	Baseline	11.1 ± 1.6	11.2 ± 1.6
				3 m post	7.9 ± 1.6	12.1 ± 1.6
				12 m post	N/A	N/A
Stair test	McKay et al., 2012	Stair ascent/descent	Seconds	Baseline	34.53 ± 29.51	33.31 ± 27.42
				3 m post	26.99 ± 26.73	22.18 ± 10.98
				12 m post	N/A	N/A
Knee extension strength	Skoffer et al., 2016, 2020	Isometric knee extension strength	Nm/kg	Baseline	1.0 ± 0.3	1.0 ± 0.4
				3 m post	1.4 ± 0.4	1.3 ± 0.5
				12 m post	1.40 ± 0.3	1.3 ± 0.4
Knee extension strength	Hermann et al., 2015; Holsgaard-Larsen et al., 2020	Isometric knee extension strength	Nm	Baseline	90.9 ± 34.5	89.4 ± 36.7
				3 m post	105.7 ± 40.6	83.7 ± 32.6
				12 m post	106.6 ± 29.8	85.9 ± 40.4
Knee extension strength	Calatayud et al., 2016	Isometric knee extension strength	Kg	Baseline	23.5 ± 7.5	23.5 ± 7.8
				3 m post	22.8 ± 7.5	14.3 ± 7.3
				12 m post	N/A	N/A
Knee extension strength	McKay et al., 2012	Isometric knee extension strength	Nm/Kg	Baseline	0.96 ± 0.58	0.84 ± 0.52
				3 m post	0.77 ± 0.56	0.74 ± 0.35
				12 m post	N/A	N/A
Knee extension strength	Domínguez-Navarro et al., 2021	Isometric knee extension strength	*N*	Baseline	99.7 ± 29.7	101.8 ± 25.5
				3 m post	N/A	N/A
				12 m post	158.3 ± 67.2	128.3 ± 32.7
Pain	Skoffer et al., 2016, 2020	KOOS pain	0–100	Baseline	53.0 ± 13.3	53.4 ± 13.5
				3 m post	78.1 ± 16.3	79.9 ± 14.2
				12 m post	89.9 ± 13.2	89.0 ± 10.1
Pain	Hermann et al., 2015; Holsgaard-Larsen et al., 2020	HOOS pain	0–100	Baseline	48.0 ± 12.7	46.3 ± 14.4
				3 m post	86.8 ± 15.6	81.4 ± 16.4
				12 m post	87.0 ± 16.5	85.5 ± 20.6
Pain	Calatayud et al., 2016	WOMAC pain	0–100	Baseline	10.6 ± 1.0	10.5 ± 1.0
				3 m post	2.9 ± 1.0	3.8 ± 1.0
				12 m post	N/A	N/A
Pain	McKay et al., 2012	WOMAC pain	0–100	Baseline	10.80 ± 2.20	11.92 ± 3.58
				3 m post	4.40 ± 3.20	3.58 ± 4.40
				12 m post	N/A	N/A
Pain	Domínguez-Navarro et al., 2021	KOOS pain	0–100	Baseline	54.9 ± 14.9	49.2 ± 13.6
				3 m post	N/A	N/A
				12 m post	92.2 ± 5.7	88.7 ± 7.8
Symptoms	Skoffer et al., 2016, 2020	KOOS symptoms	0–100	Baseline	60.1 ± 17.7	59.0 ± 18.7
				3 m post	72.8 ± 16.4	71.9 ± 11.4
				12 m post	86.5 ± 13.1	83.4 ± 14.5
Symptoms	Hermann et al., 2015; Holsgaard-Larsen et al., 2020	HOOS symptoms	0–100	Baseline	44.5 ± 16.4	43.1 ± 18.5
				3 m post	79.9 ± 15.0	74.6 ± 18.6
				12 m post	79.6 ± 16.9	83.4 ± 20.6
Symptoms	Domínguez-Navarro et al., 2021	KOOS symptoms	0–100	Baseline	64.1 ± 14.3	64.6 ± 12.6
				3 m post	N/A	N/A
				12 m post	93.4 ± 7.4	91.4 ± 9.9
Activities of daily living	Skoffer et al., 2016, 2020	KOOS ADL	0–100	Baseline	53.0 ± 13.3	53.4 ± 13.5
				3 m post	72.8 ± 16.4	71.9 ± 11.4
				12 m post	87.6 ± 12.3	84.4 ± 11.8
Activities of daily living	Holsgaard-Larsen et al., 2020	HOOS ADL	0–100	Baseline	49.2 ± 12.5	48.1 ± 13.8
				3 m post	79.9 ± 15.0	74.6 ± 18.6
				12 m post	86.5 ± 13.8	82.5 ± 23.0
Activities of daily living	Domínguez-Navarro et al., 2021	KOOS ADL	0–100	Baseline	55.5 ± 17.8	51.7 ± 11.7
				3 m post	N/A	N/A
				12 m post	88.1 ± 6.8	87.8 ± 4.6
Sport & recreation	Skoffer et al., 2016, 2020	KOOS sport & recreation	0–100	Baseline	23.7 ± 16.7	20.2 ± 19.9
				3 m post	50.2 ± 28.4	40.0 ± 22.5
				12 m post	59.5 ± 27.5	55.0 ± 18.4
Sport & Recreation	Hermann et al., 2015; Holsgaard-Larsen et al., 2020	HOOS sport & recreation	0–100	Baseline	28.1 ± 15.2	27.8 ± 17.7
				3 m post	73.8 ± 19.8	62.4 ± 24.7
				12 m post	75.3 ± 20.4	68.5 ± 31.6
Quality of life	Skoffer et al., 2016, 2020	KOOS QOL	0–100	Baseline	39.6 ± 14.8	33.8 ± 14.4
				3 m post	66.2 ± 18.9	61.9 ± 16.6
				12 m post	78.6 ± 19.1	73.4 ± 15.2
Quality of life	Hermann et al., 2015; Holsgaard-Larsen et al., 2020	HOOS QOL	0–100	Baseline	32.1 ± 14.4	29.2 ± 15.6
				3 m post	74.6 ± 18.4	70.3 ± 23.1
				12 m post	75.3 ± 20.4	74.0 ± 30.2
Quality of life	Domínguez-Navarro et al., 2021	KOOS QOL	0–100	Baseline	31.8 ± 12.2	28.3 ± 12.2
				3 m post	N/A	N/A
				12 m post	71.4 ± 8.9	67.6 ± 9.2

*SD, standard deviation; 3 m post, 3 months post-operatively; 12 m post, 12 months post-operatively; N/A, not available; KOOS, Knee disability & osteoarthritis outcome score; HOOS, Hip disability and osteoarthritis outcome score; ADL, activities of daily living; QOL, quality of life*.

### Risk of Bias Assessement and Grade Assessment

RoB assessments for all included trials are presented in Figure 1. RoB was judged low for Hermann et al. (2015) and Skoffer et al. (2016). Some concerns were noted with regard to the randomization process, the selection of reported results, and missing information on pre-registration in Calatayud et al. (2016). Likewise, concerns regarding the randomization process was noted for Domínguez-Navarro et al. (2021), along with high risk of bias with regard to missing outcome data. Finally, concerns with regards to deviations from the intended intervention procedures, missing outcome data, measurements of outcome variables, and selection of reported results were noted for McKay et al. (2012).

The level of certainty in evidence was rated low-to-very low for all outcome variables, mainly due to moderate-to-high risks of bias (Table 3).

**Table 3:** Meta-analysis results.

**Outcomes**	**SMD [95% CI]**		**Number of Participants (studies)**	**Quality of evidence**
	**Exercise intervention**			
**3 months post**				
Sit to stand	ES = 0.74	[0.39, 1.08]	139 (2)	Low ⊕⊕□□^a^^,^^c^
Timed up and go	ES = −1.19	[−2.63, 0.25]	109 (2)	Low ⊕⊕□□^a^^,^^c^
Walking speed	ES = −0.51	[−0.99, −0.09]	106 (3)	Very low ⊕□□□^a^^,^^b^^,^^c^
Stair climbing	ES = −1.15	[−2.58, 0.29]	147 (3)	Very low ⊕□□□^a^^,^^b^^,^^c^
Knee extension strength	ES = 0.55	[0.08, 1.02]	206 (4)	Low ⊕⊕□□^a^^,^^c^
Knee flexion strength	ES = 1.95	[−1.11, 5.02]	109 (2)	Low ⊕⊕□□^a^^,^^c^
Pain	ES = 0.30	[−0.14, 0.75]	206 (4)	Low ⊕⊕□□^a^^,^^c^
Symptoms	ES = 0.20	[−0.15, 0.56]	139 (2)	Very low ⊕□□□^a^^,^^b^^,^^c^
ADL	ES = 0.41	[0.08, 0.75]	139 (2)	Low ⊕⊕□□^a^^,^^c^
Sport and recreation	ES = 0.46	[0.12, 0.80]	139 (2)	Low ⊕⊕□□^a^^,^^c^
Quality of life	ES = 0.27	[−0.07, 0.61]	139 (2)	Low ⊕⊕□□^a^^,^^c^
**12 months post**				
Sit to stand	ES = 0.51	[0.14, 0.88]	117 (2)	Very low ⊕□□□^a^^*^^,^^c^
Timed up and go	ES = −0.20	[−0.64, 0.24]	84 (2)	Very low ⊕□□□^a^^,^^b^^,^^c^
Walking speed	ES = −0.37	[−0.75, 0.00]	117 (2)	Very low ⊕□□□^a^^*^^,^^c^
Knee extension strength	ES = 0.48	[0.15, 0.82]	152 (3)	Low ⊕⊕□□^a^^,^^c^
Pain	ES = 0.39	[0.02, 0.77]	147 (3)	Very low ⊕□□□^a^^*^^,^^c^
Symptoms	ES = −0.01	[−0.44, 0.42]	147 (3)	Very low ⊕□□□^a^^*^^,^^c^
ADL	ES = 0.19	[−0.14, 0.51]	147 (3)	Very low ⊕□□□^a^^*^^,^^c^
Sport and recreation	ES = 0.23	[−0.14, 0.60]	112 (2)	Low ⊕⊕□□^a^^,^^c^
Quality of life	ES = 0.22	[−0.13, 0.56]	106 (3)	Low ⊕⊕□□^a^^,^^c^

*Certainty and quality of evidence. CI, confidence interval; ES, effect size; SMD, standardized mean difference*.

* *, downgraded two steps*:

a *Downgraded due to risk of bias*;

b *Downgraded due to inconsistency*;

c *Downgraded due to imprecision*.

### Effects of Prehabilitation vs. Standard Care or Control Intervention on Functional Performance, Knee Extensor and Flexor Strength, and Patient-Reported Outcomes

A total of 6 studies were included in the meta-analyses. We conducted seven meta-analyses comparing prehabilitation with usual care or control intervention 3 months post-operative on sit-to-stand performance (Skoffer et al., 2016; Holsgaard-Larsen et al., 2020), Timed Up&Go (Calatayud et al., 2016; Skoffer et al., 2016), walking speed (McKay et al., 2012; Skoffer et al., 2016; Holsgaard-Larsen et al., 2020), stair climbing (McKay et al., 2012; Calatayud et al., 2016; Holsgaard-Larsen et al., 2020), and 12 months post-operative for sit-to-stand performance (Holsgaard-Larsen et al., 2020; Skoffer et al., 2020), Timed Up & Go (Skoffer et al., 2020; Domínguez-Navarro et al., 2021), and walking speed (Holsgaard-Larsen et al., 2020; Skoffer et al., 2020). We conducted three meta-analyses comparing the effect of prehabilitation with usual care or control intervention on knee extensor 3 months post-operatively (McKay et al., 2012; Calatayud et al., 2016; Skoffer et al., 2016; Holsgaard-Larsen et al., 2020), knee flexor strength 3 months post-operatively (Calatayud et al., 2016; Skoffer et al., 2016), and knee extensor strength 12 months post-operatively (Holsgaard-Larsen et al., 2020; Skoffer et al., 2020; Domínguez-Navarro et al., 2021); and ten meta-analyses on pain assessed at 3 months (McKay et al., 2012; Calatayud et al., 2016; Skoffer et al., 2016) and 12 months post-operatively (Holsgaard-Larsen et al., 2020; Skoffer et al., 2020; Domínguez-Navarro et al., 2021), symptoms 3 months (Skoffer et al., 2016; Holsgaard-Larsen et al., 2020) and 12 months post-operatively (Holsgaard-Larsen et al., 2020; Skoffer et al., 2020; Domínguez-Navarro et al., 2021), activities of daily living 3 months (Skoffer et al., 2016; Holsgaard-Larsen et al., 2020) and 12 months post-operatively (Holsgaard-Larsen et al., 2020; Skoffer et al., 2020; Domínguez-Navarro et al., 2021), Sport & Recreation 3 months post-operatively (Skoffer et al., 2016; Holsgaard-Larsen et al., 2020), and quality of life 3 months (Skoffer et al., 2016) and 12 months post-operatively (Holsgaard-Larsen et al., 2020; Skoffer et al., 2020; Domínguez-Navarro et al., 2021).

**Figure 3:**
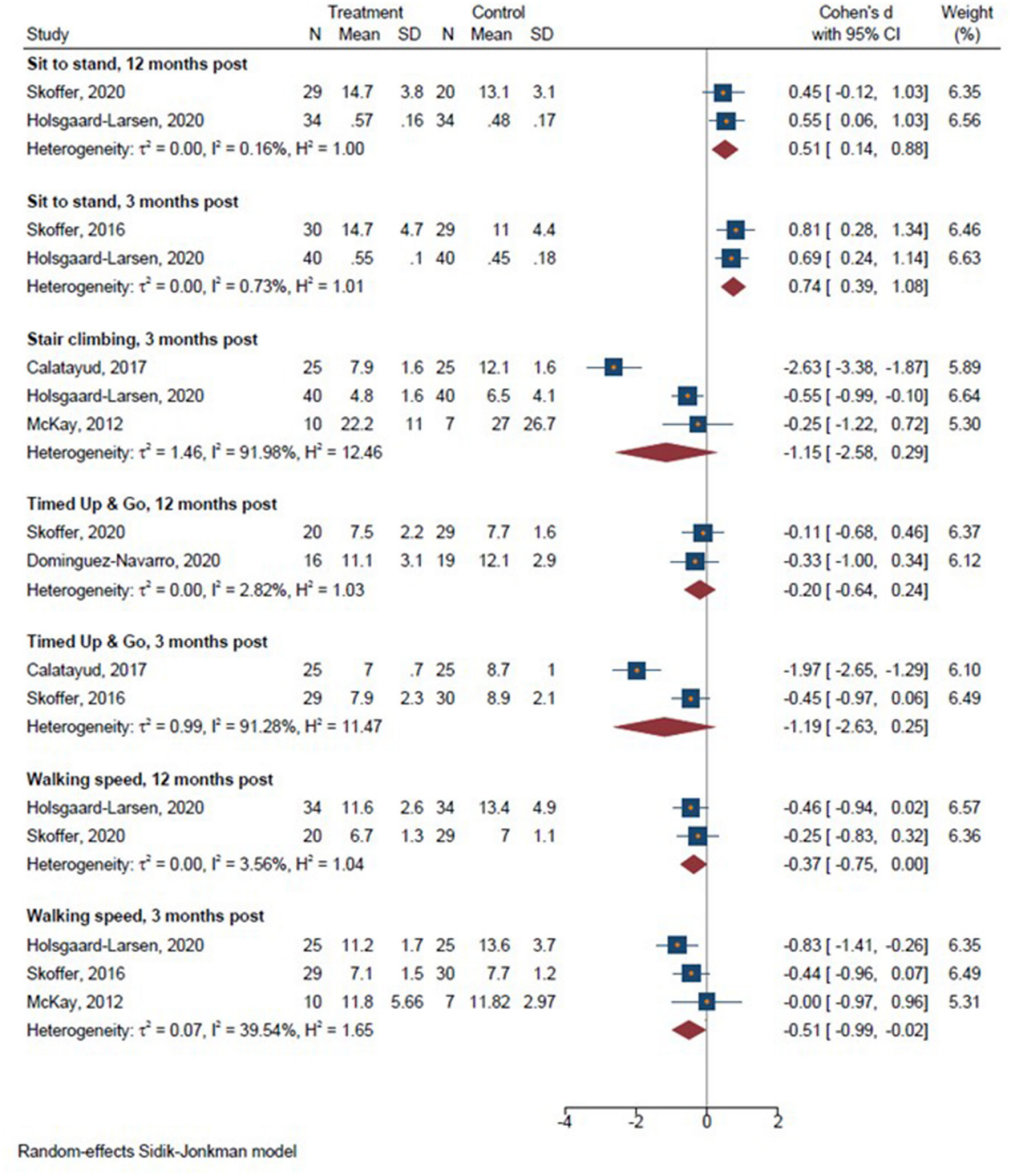
Forrest plots on post-operative functional performance 3 and 12 months post-operatively. Forest plots of the results of a random-effects meta-analysis shown as standardized mean differences with 95% CIs on functional performance 3 and 12 months post-operatively. For each study, the blue square represents the point estimate of the intervention effect. The horizontal line joins the lower and upper limits of the 95% CI of this effect. The red diamonds represent the pooled mean difference for each outcome.

**Figure 4:**
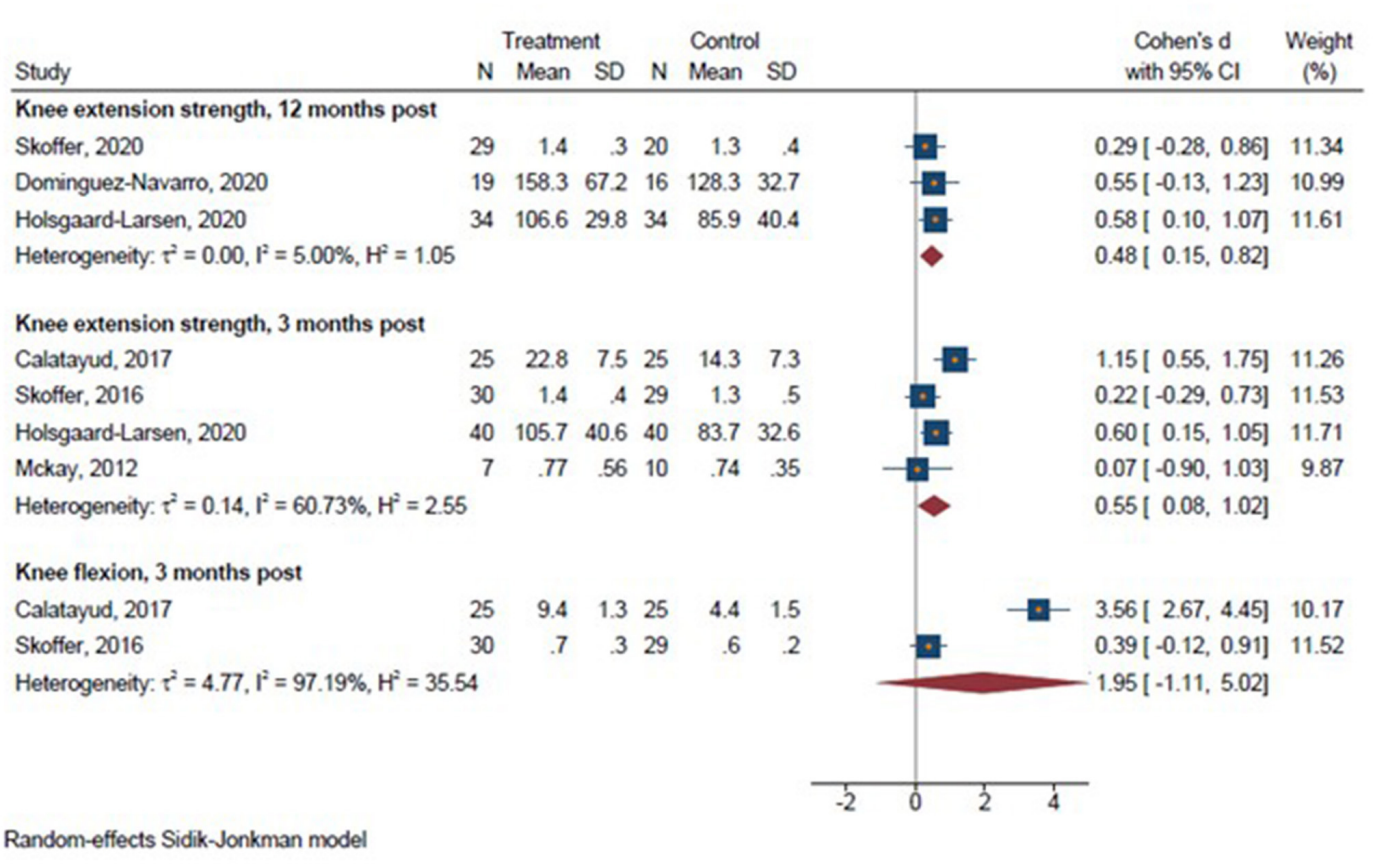
Forrest plots on lower limb strength 3 and 12 months post-operatively. Forest plots of the results of a random-effects meta-analysis shown as standardized mean differences with 95% CIs on lower limb strength 3 and 12 months post-operatively. For each study, the blue square represents the point estimate of the intervention effect. The horizontal line joins the lower and upper limits of the 95% CI of this effect. The red diamonds represent the pooled mean difference for each outcome.

**Figure 5:**
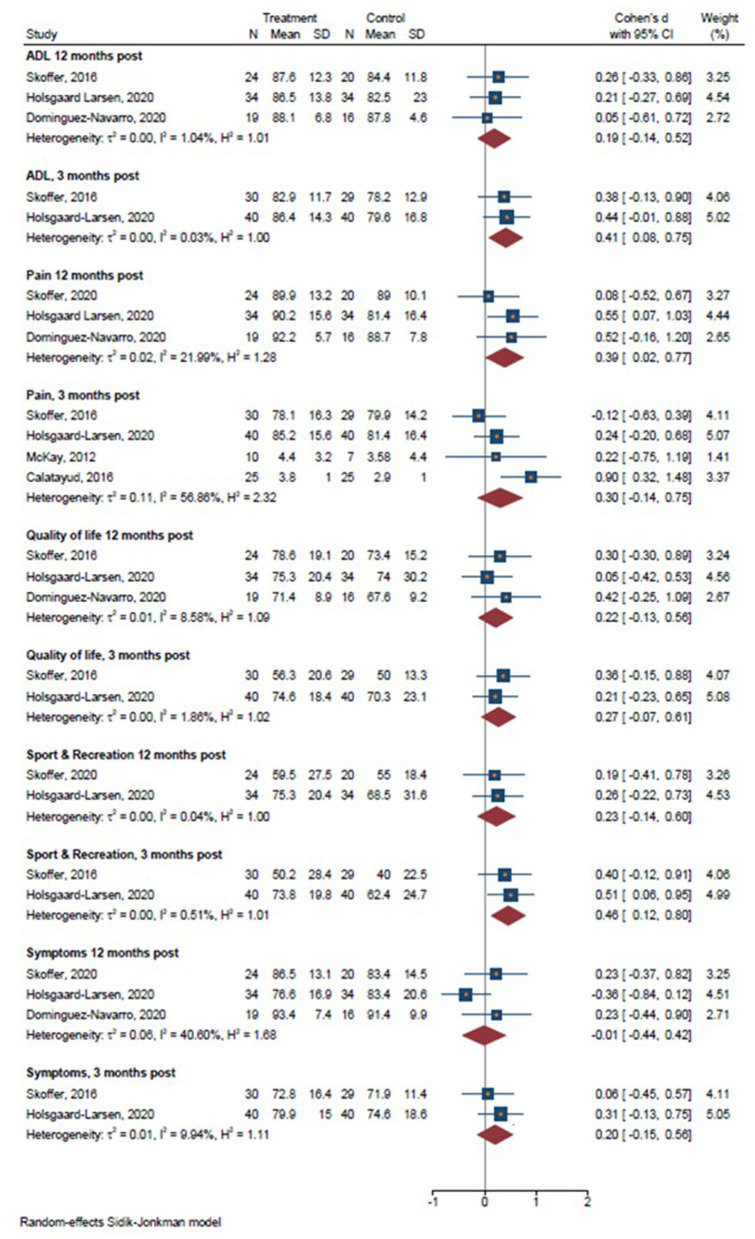
Forrest plots on patient-reported outcomes 3 and 12 months post-operatively. Forest plots of the results of a random-effects meta-analysis shown as standardized mean differences with 95% CIs on patient-reported outcomes 3 and 12 months post-operatively. For each study, the blue square represents the point estimate of the intervention effect. The horizontal line joins the lower and upper limits of the 95% CI of this effect. The green diamonds represent the pooled mean difference for each outcome.

There was a significant effect in favor of prehabilitation on sit-to-stand performance 3 and 12 months post-operatively, on walking speed 3 and 12 months post-operatively, while no significant effect favoring prehabilitation for Timed Up & Go and stair climbing or stair climbing performance (Figure 3) (Holsgaard-Larsen et al., 2020; Skoffer et al., 2020; Domínguez-Navarro et al., 2021). Furthermore, a significant effect in favor of prehabilitation on maximal knee extensor strength emerged 3 and 12 months post-operatively, whereas no significant effect in favor of prehabilitation was observed for knee flexor strength 3 months post-operatively (Figure 4). Lastly, a significant effect in favor of prehabilitation was observed for ADL 3 months post-operatively, Sport & Recreation 3 months post-operatively, and pain 12 months post-operatively. No effect in favor of prehabilitation was found for the remaining patient-reported outcomes (Figure 5).

## Discussion

The main finding of the present meta-analysis was that pre-operative prehabilitation training involving progressive resistance training (PRT) prior to TJR was indeed effective in producing enhanced medium-term and long-term gains in sit-to-stand performance, horizontal gait speed, and maximal knee extensor muscle strength compared to usual are or control intervention. Also, accentuated improvements in patient-reported outcomes representing the perceived ability to engage in activities of daily living, sport and recreational activities, along with larger reductions in pain were observed with PRT-based prehabilitation training up to 12 months post-operatively. However, not all functional performance measures or patient-reported outcomes were favored by PRT prior to TJR. The heterogeneous observations between different studies may in part rely on differences in total training volume and/or loading intensity (cf. Table 3). Thus, a pronounced degree of methodological diversity appears to exist between study specific exercise protocols, which may help to explain the marked differences in effects sizes observed across studies (cf. Figure 3). Specifically, McKay et al. (2012) showed no difference between groups for functional performance 3 months after surgery while Calatayud et al. (2016) and Holsgaard-Larsen et al. (2020) demonstrated significant differences between groups for the same outcomes (Figure 3). Thus, it may appear that mirroring a training protocol to Calatayud et al. (2016) or Hermann et al. (2015) would result in higher post-operative gains in functional performance and lower limb strength.

Thus, interpreting the singular results in i.e., Figure 3 from a clinical perspective, it appears that adopting a training protocol similar to Calatayud et al. (2016) or Hermann et al. (2015) may result in more pronounced post-operative gains in functional performance and lower limb muscle strength, respectively.

No previous systematic review has been able to identify any long-term effects of prehabilitation on various measures of objective functional performance or maximal knee extensor strength after TJR. This lack of identifiable effects may, at least in part, be ascribed to the inclusion of intervention protocols that are suboptimal for improving skeletal muscle strength. Thus, the inclusion of study trials utilizing unknown-to-low loading intensities and/or total training volumes and/or submaximal exercise protocols may have contributed to dilute the sensitivity of the overall meta-analysis to document the true effect of prehabilitation activities based on more optimized resistance training paradigms with documented anabolic (Aagaard et al., 2001) and neuro-facilitating (Aagaard et al., 2002) effects.

In the present meta-analysis, patient-reported *ADL* and *Sport & Recreation* were positively affected by prehabilitation exercise training when assessed 3 months after surgery (moderate effect). Furthermore, a small effect favoring prehabilitation was found for patient-reported pain 12 months after surgery. Hence, the present meta-analysis points to positive effects in both objectively measured function and patient reported function up to 12 months after surgery, in contrast to previous meta-analyses (Moyer et al., 2017).

The effect of prehabilitation on patient reported outcomes was small yet clearly evident in the present meta-analysis. The attenuated effect on patient reported outcomes may be explained by patients undergoing TJR achieve a very large perceived improvement from the surgical procedure (illustrated in Table 2). Hence, the range of subjectively perceived improvements imposed by exercise may be limited in such patients. Nonetheless, we were able to demonstrate a facilitating effect of prehabilitation training on this parameter in the present meta-analysis, which is a notable finding given the relatively small overall sample size (*n* = 256).

All of the included trials as well as one ongoing trials (Jørgensen et al., 2020) utilized fully supervised exercise session throughout the entire intervention period. Future studies should be conducted to examine if exercise protocols involving less extensive 1:1 supervision will be able to ensure a high adherence to training as well as a sufficient (i.e., effective) quality of exercise. This would likely facilitate the implementation of pre-operative training into the healthcare systems and offer more patients the opportunity to improve key outcome parameters (i.e., lower-limb strength and functional performance) associated with a higher post-operative functional performance-level in a “better in, better out”-manner.

### Strengths and Limitations

In terms of methodological strengths, the present study adhered to the guidelines outlined by the Cochrane Handbook for Systematic Reviews of Interventions [version 6.2 (updated February 2021)] and the PRISMA statement (Moher et al., 2015). Specifically, inclusion and exclusion criteria were stated a priori, while study populations were comparable across trials and a majority of the functional performance tests remained similar across trials. As an additional strength of the present study, all included trials reported data on the specific exercise intensity, to ensure that sufficient exercise intensity and volume were employed in all studies included in the analyses.

A number of limitations may exist with the present meta-analysis. Firstly, the low number of studies (*n* = 7) included in the present systematic review may be considered a limitation, especially since comprising only five independent trials. However, as only RCT studies with relatively similar populations were included, and a random effects model with an adjustment to the confidence interval due few eligible studies (IntHout et al., 2014) was applied, we consider the results of the present analyses robust and valid. Despite being unable to perform our preplanned search in the The Allied and Complementary Database, we deem that the present literature search was effective of capturing all relevant studies.

Secondly, only a single study investigated the effect of pre-operative PRT in patients scheduled for THR, thus limiting the generalizability of the involved sub-analysis to patients suffering from end-stage hip OA. However, despite that TKR and THR are inherently different surgeries with differing effects on muscle and functional performance, and with different trajectories of recovery, it has been proposed that both patient populations may benefit from improving functional performance and lower-limb muscle strength prior to surgery (Bade et al., 2010; Zeni and Snyder-Mackler, 2010; Holstege et al., 2011; Nankaku et al., 2013; Skoffer et al., 2015). Therefore, before any firm conclusions can be drawn on the benefits of pre-operative PRT for patients scheduled for THR on post-operative functional performance, lower limb strength, and patient-reported outcomes, more research on this particular patient population is warranted.

Thirdly, despite exclusively including studies using exercise modalities and loading intensities objectively known to increase muscle strength and mass, intervention protocols were found to differ markedly between studies in terms of duration, total training volume and loading intensity. Consequently, optimal prehabilitation exercise dosage in terms of loading intensity and total duration remains to be investigated in patients scheduled for TJR surgery.

Fourthly, only very few studies have examined the long-term effects of strength-based prehabilitation in TJR patients (Holsgaard-Larsen et al., 2020; Skoffer et al., 2020; Domínguez-Navarro et al., 2021), underlining the need for more research to confirm the conclusions of the present meta-analysis. Furthermore, due to relatively high dropout rates from baseline to 12-month follow-up (Holsgaard-Larsen et al., 2020; Skoffer et al., 2020; Domínguez-Navarro et al., 2021), it appears important to ensure that future RCTs are sufficiently powered to detect long-term effects (≥12 months).

Lastly, low-to-very-low quality evidence formed all the comparisons in this systematic review. Our certainty of evidence was downgraded due to limitations in the randomization process, mainly due to deviations from the intended intervention procedures, missing data, and selection of the reported results. However, due to the nature of RCTs involving an exercise intervention group vs. usual care or control intervention group, in nature preventing from achieving full blinding of all participants and observers, it seems impossible to achieve high-level evidence when applying the GRADE assessment tool.

## Conclusions

The present meta-analyses demonstrates that prehabilitation training involving progressive resistance exercise prior to TJR effectively induce long-lasting improvements in functional performance, maximal knee extensor muscle strength, and pain scoring, respectively. However, due to large methodological heterogeneity between the exercise protocols applied in the present studies, optimal choices about loading intensities, duration and total training volume remains unknown.

## Data Availability Statement

The original contributions presented in the study are included in the article/Supplementary Material, further inquiries can be directed to the corresponding author/s.

## Author Contributions

SJ and SK organized the database and selected the appropriate studies. MB and SJ performed the data exctraction. SJ performed the statistical analysis and wrote the first draft of the manuscript. All authors contributed to conception, design of the study, participated in the data extraction, quality assessment, manuscript revision, read, and approved the submitted version.

## Funding

This study was funded by the Nis Hanssens Mindeslegat 464000 dkr, Aase & Ejnar Danielsens Foundation 250000 dkr, Central Denmark Regional Health Science Foundation 99568 dkr, The Association for Danish Physiotherapists Foundation for research 131600 dkr, Hede Nielsens Foundation 58199 dkr, Hartmann's Foundation 94049 dkr.

## Conflict of Interest

The authors declare that the research was conducted in the absence of any commercial or financial relationships that could be construed as a potential conflict of interest.

## Publisher's Note

All claims expressed in this article are solely those of the authors and do not necessarily represent those of their affiliated organizations, or those of the publisher, the editors and the reviewers. Any product that may be evaluated in this article, or claim that may be made by its manufacturer, is not guaranteed or endorsed by the publisher.
